# Proteoglycans in Obesity-Associated Metabolic Dysfunction and Meta-Inflammation

**DOI:** 10.3389/fimmu.2020.00769

**Published:** 2020-05-19

**Authors:** Ariane R. Pessentheiner, G. Michelle Ducasa, Philip L. S. M. Gordts

**Affiliations:** ^1^Department of Medicine, Division of Endocrinology and Metabolism, University of California, San Diego, La Jolla, CA, United States; ^2^Glycobiology Research and Training Center, University of California, San Diego, La Jolla, CA, United States

**Keywords:** diabetes, extracellular matrix, metabolic inflammation, obesity, proteoglycans

## Abstract

Proteoglycans are a specific subset of glycoproteins found at the cell surface and in the extracellular matrix, where they interact with a plethora of proteins involved in metabolic homeostasis and meta-inflammation. Over the last decade, new insights have emerged on the mechanism and biological significance of these interactions in the context of diet-induced disorders such as obesity and type-2 diabetes. Complications of energy metabolism drive most diet-induced metabolic disorders, which results in low-grade chronic inflammation, thereby affecting proper function of many vital organs involved in energy homeostasis, such as the brain, liver, kidney, heart and adipose tissue. Here, we discuss how heparan, chondroitin and keratan sulfate proteoglycans modulate obesity-induced metabolic dysfunction and low-grade inflammation that impact the initiation and progression of obesity-associated morbidities.

## Introduction

Obesity and its co-morbidities are responsible for a global health problem carrying a significant economic burden. The most common obesity-mediated complications include type-2 diabetes (T2D), cardiovascular disease and chronic kidney disease, yet the contributing mechanisms to these diseases remain to be fully established (1, 2). Obesity and in particular central adiposity – the excess deposition of visceral fat – is associated with increased serum levels of pro-inflammatory cytokines such as interleukin 6 (IL-6), C-reactive protein (CRP), and tumor necrosis factor (TNF) (3–5). This type of low-grade tissue inflammation, also called meta-inflammation, of multiple organs such as liver, adipose tissue, pancreas, kidney, heart, and brain is an important contributing risk factor for the development of insulin resistance. The ensuing chronic tissue inflammation is also associated with fibrosis and necrosis, leading to progressive tissue damage. While obesity-associated inflammation is well recognized, the exact etiology is still poorly understood. The complex nature of obesity-induced inflammation presents a challenge to understand the underlying molecular mechanisms that contribute to development of obesity and its associated inflammation. The extracellular matrix (ECM) surrounding cells is a central hub in mediating metabolic and inflammatory signal transduction, regulating fibrotic processes and ensuring the functional integrity of cells. In this review we will describe the central role of heparan, chondroitin and keratan sulfate proteoglycans found in the ECM in processes critical for initiation, progression and the chronic nature of meta-inflammation in the context of obesity and T2D.

## Diet-Induced Meta-Inflammation Affects Specific Tissues Critical for Energy Homeostasis

### Adipose Tissue Inflammation

Adipose tissue (AT) has an enormous plasticity to adapt to nutritional changes. However, under conditions of constant overnutrition, AT expands beyond its limits and is associated with the development of inflammation, impaired angiogenesis and ectopic fat deposition in organs such as liver and skeletal muscle. This detrimental cycle leads to tissue dysfunction and development of insulin resistance. Adipose depots are a conglomerate of various cell types, such as mature adipocytes embedded in the stroma with preadipocytes, fibroblasts, immune cells and endothelial cells. Immune cells, primarily macrophages, residing in AT maintain the integrity and hormonal sensitivity of adipocytes in a normal lean state. Macrophages are characterized by their “polar” state, displaying more of a pro-inflammatory or anti-inflammatory and, resolving phenotype. Resident macrophages of lean subjects present an M2-polarized or resolving state which is generally associated with anti-inflammatory properties. However, during progressive weight gain and development of obesity macrophages change into pro-inflammatory M1-like macrophages, which produce pro-inflammatory cytokines attracting more M1-macrophages to infiltrate the AT. The thereby activated inflammatory program has an overall T-helper type 1 (Th1) nature, which is usually associated with infection. Unlike acute inflammation, the meta-inflammation in an obese state is not resolved, which leads to a chronic inflammatory response that triggers insulin resistance of adipocytes resulting in lipid spill-over to peripheral organs (5, 6).

### Liver Is a Central Hub for Energy Homeostasis

One of the major organs affected by diet-induced meta-inflammation and AT insulin resistance is the liver. It plays a central role in maintaining whole body energy homeostasis and is considered an energy storage and redistribution node. Consisting predominantly of hepatocytes it is also populated by fenestrated sinusoidal endothelium and Kupffer-cells, which are specific resident hepatic macrophages important for normal liver function especially in the lean state. However, in the context of energy excess, such as obesity, Kupffer-cells get activated analogous to resident AT macrophages thereby recruiting more immune cells and promoting hepatic insulin resistance. Consequently, this negatively affects insulin-mediated inhibition of hepatic glucose production (gluconeogenesis) thereby driving elevated glucose levels in afflicted patients (7, 8). Hepatic insulin resistance also prevents insulin-mediated inhibition of production of very-low density lipoproteins (VLDL) in hepatocytes and impairs hepatic lipoprotein clearance of low-density lipoproteins (LDL) and triglyceride-rich lipoprotein (TRL) remnants. This results in the onset of metabolic dyslipidemia, which in conjunction with chronic inflammation drives the progression of cardiovascular disease. Importantly, insulin resistance favors the development of excessive fat accumulation in the liver, also called steatohepatitis creating a vicious cycle of escalating inflammation, insulin resistance and fibrosis which leads to non-alcoholic fatty liver disease (NAFLD). Furthermore, AT-derived circulating cytokines and adipokines promote NAFLD (9–11). Without intervention NAFLD progresses to liver cirrhosis and ultimately to hepatocellular carcinoma. However, even after weight loss intervention, a permanent inflammatory fingerprint lingers in AT and liver (12).

### Kidney Dysfunction Induced by Meta-Inflammation

While the consequences of obesity and insulin resistance in liver and AT are often discussed, less attention is paid to the kidneys which are important for detoxifying processes and contribute to organ dysfunction in disease states. Increased obesity prevalence has been associated with a rise in chronic kidney disease progression and not surprisingly obese individuals are at elevated risk for developing end-stage kidney disease (13). Similarly, experimental mouse models of obesity, such as high fat diet (HFD) fed models, develop increased kidney injury (14). HFD-mediated kidney injury results in leakage of albumin in the urine (albuminuria), renal fibrosis, insulin resistance, and elevated inflammation in mice (15). Obesity and diabetes mediated end-stage kidney disease is characterized by an accumulation of lipids within the kidney met with increased inflammation (2). IL-6, TNF, IL-1β and MCP-1 levels are elevated in kidneys from a diet-induced obesity rat model, associated with increased kidney fibrosis and sclerotic lesions (16). Similarly, TNF is elevated in sera obtained from patients with diabetic kidney disease (DKD), as well as in an obese-diabetic mouse model that presents with kidney disease (17). Furthermore, oxidative stress is upregulated in kidneys from preclinical obesity-induced DKD models (18) together with increased inflammation in perirenal visceral AT, all of which was reversed after treatment with an angiotensin II inhibitor (ANG 1-7) (19). Collectively, these data suggest that obesity and AT inflammation contribute to kidney complications observed in obese experimental and clinical models.

## The Extracellular Matrix Surrounding Adipocytes Plays a Central Role in Metabolic Inflammation

In recent years the importance of the adipocyte microenvironment has gained more prominence as ECM composition, remodeling and interacting factors significantly contribute to the detrimental consequences of obesity. The ECM is a key regulator for maintaining optimal cell and tissue homeostasis by disseminating and integrating cues from and to surrounding cells as well as distant organs. ECM remodeling is critical for differentiation of adipocytes, the integrity of expanding adipocytes as well as recruitment of immune cells (20). AT has the capacity to expand either through hyperplasia, a result of increased preadipocyte proliferation and differentiation into adipocytes, or through hypertrophy by expanding the lipid storage capacity of existing adipocytes. In the process of adipocyte differentiation, the ECM undergoes structural changes from a fibrillar to a laminar structure. The fibrillar structure of preadipocytes, mainly containing collagen I, plasmin, and fibronectin, is replaced by a laminar structure built by collagen VI, laminin, and a high amounts of collagen IV (21). During excessive AT expansion, imbalances in ECM synthesis and degradation lead to fibrosis, one of the hallmarks of AT dysfunction associated with meta-inflammation and progression of advanced insulin resistance (22). Moreover, hypertrophy induces hypoxia and mechanical stress (6, 23, 24). Hypoxia contributes to meta-inflammation through activation of hypoxia inducible factor 1 (HIF1) resulting in increased transcription of a pro-inflammatory gene program in adipocytes and patrolling immune cells (23, 24). Concomitantly, angiogenesis is induced during AT expansion to support the growing tissue with essential nutrients, hormones, growth factors, and oxygen. However, in an obese state, the angiogenic capacity of endothelial cells declines, resulting in augmented tissue hypoxia and cell apoptosis. This process requires infiltration of immune cells including macrophages to clear dying adipocytes thereby forming so-called crown-like structures. Although flexible to some degree, the ECM provides a rigid mesh that puts mechanical pressure on adipocytes. This occurs during hypertrophy and induces an inflammatory signature during excessive expansion (20). Modulating that mechanical stress by targeting collagen VI levels improves lipid and energy metabolism in adipocytes and attenuates fibrosis and metabolic inflammation (25). These observations emphasize the therapeutic potential of ECM modulation.

## Proteoglycans Are Ubiquitous Extracellular Environment Components Regulating Numerous Metabolic Processes

**FIGURE 1:**
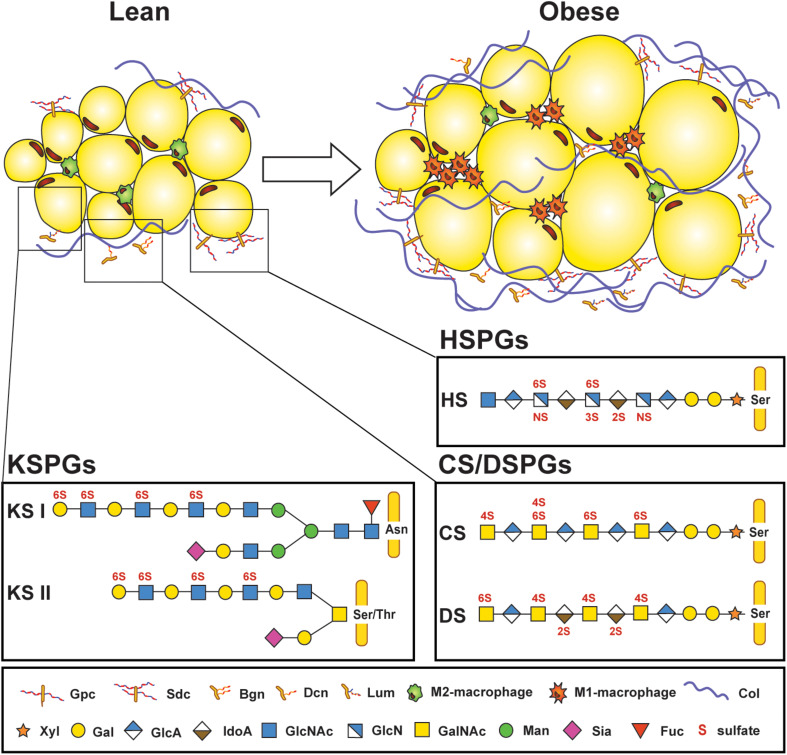
Proteoglycans are part of the microenvironment surrounding adipocytes. Lean adipose tissue expresses low amounts of proteoglycans found in the extracellular matrix surrounding adipocytes and stromal vascular cells, but their expression increases in obese adipose tissue, along with increased infiltration of M1-macrophages and deposition of collagen. Several core proteins are linked to adipose tissue homeostasis, such as glypican (Gpc) and syndecans (Sdc); biglycan (Bgn), decorin (Dcn), and lumican (Lum). Three different types of glycosaminoglycan (GAG) chains that are covalently attached to protein cores can be found in adipose tissue. Keratan sulfate (KS) chains are either attached to asparagine (Asn) or serine/threonine (Ser/Thr) at the protein core and consist of repeating *N*-acetylglucosamine (GlcNAc) and galactose (Gal) subunits. KS chains can also be sialylated (Sia) or fucosylated (Fuc). Heparan sulfate (HS) and chondroitin/dermatan sulfates (CS/DS) are attached through a tetrasaccharide linker region starting with a xylose (Xyl), two Gal, and a glucuronic acid (GlcA) at a Ser on the protein core. HS consists of *N*-acetyl-glucosamine (GlcNAc) and a combination of either GlcA or iduronic acid (IdoA). CS features *N*-acetyl galactosamine (GalNAc) and GlcA residues which can be replaced by IdoA in DS. GAG chains are sulfated on various positions (*N*-,2-*O*, 3-*O*, 4-*O*, or 6-*O*-sulfations depicted in red). Col: Collagen. [Proteoglycan structures adapted from Ref. (26)].

Proteoglycans (PGs) are an integral part of the cellular glycocalyx in the ECM and exhibit important roles in cell and tissue homeostasis by regulating various processes such as proliferation, differentiation, angiogenesis, and inflammation. They consist of a core protein with one or more covalently attached glycosaminoglycan (GAG) chains (Figure 1). The building block of those GAG chains are repeating disaccharide units consisting of an amino sugar and a uronic acid which depending on the alternating glycan units determine the class of proteoglycans. Keratan sulfate (KS) does not contain a uronic acid but is built of repeating units of galactose (Gal) and *N*-Acetyl-glucosamine (GlcNAc) attached to *N*- (KS-I) or *O*-glycan (KS-II) chains (Figure 1). Chondroitin sulfate (CS) consists of repeating units of *N-*acetyl-D-galactosamine (GalNAc) and glucuronic acid (GlcA) residues (Figure 1). Dermatan sulfate differs from CS only by the additional presence of iduronic acids (IdoA) as a result of GlcA epimerization. In contrast, heparan sulfate (HS) features repeating units of GlcNAc or *N*-sulfated glucosamine (GlcNS) and a combination of either GlcA or IdoA (Figure 1). Hence, there are three main proteoglycan subclasses – chondroitin/dermatan sulfate proteoglycans (CS/DSPG), heparan sulfate proteoglycans (HSPG), and keratan sulfate proteoglycans (KSPG) (Figure 1). One important feature of GAG chains attached to proteoglycans is their high degree of sulfation resulting in generation of a strongly negatively charged polysaccharide. This gives proteoglycans an enormous capacity to act as a charge-barrier in the ECM, hindering some factors to bind and allowing others to interact (26). Many ligands of PGs have been identified over the years, including growth factors such as fibroblast growth factors (FGFs), cytokines and chemokines, cell surface receptors, cell adhesion molecules, and other ECM components such as collagen and fibronectin. Some proteoglycan interactions require GAG chains, others are depending on the core protein (27–29). Not surprisingly, proteoglycans are crucial regulators of many metabolic homeostatic processes as well as of acute and chronic inflammation (30–34). Several proteoglycans, including lumican, perlecan, decorin, aggrecan, versican, betaglycan, biglycan, and proteoglycan 4, have been described to be either secreted by adipocytes or are at least abundantly present in their ECM (20, 35). Proteoglycan composition is influenced by the diabetic condition (36), however, the amount of PGs produced during differentiation of preadipocytes to adipocytes is controversial with some studies noting decreased PG production during differentiation (37), and others reporting the opposite trend (38). The interaction of ECM components, such as collagens and proteoglycans, with cell surface receptors is crucial during the differentiation and expansion of adipocytes. Therefore, a detailed understanding of the role proteoglycans and their interaction partners play can provide novel solutions to tackle obesity-related meta-inflammation. The link between diet-induced metabolic inflammation and the development of insulin resistance has been well-established over the past years, but specific mechanisms of how the microenvironment surrounding cells influences the progression of inflammation are still under investigation. In this review, we will focus on the roles of proteoglycans and their structural modifications in metabolic homeostasis as well as their influence on AT hypertrophy and hyperplasia and their impact on meta-inflammation in critical organs for obesity-associated metabolic complications such as AT, liver and kidney.

## Heparan Sulfate Proteoglycans, a Defined Group of Glycoproteins, Act as Reostats in Metabolic Disease

**FIGURE 2:**
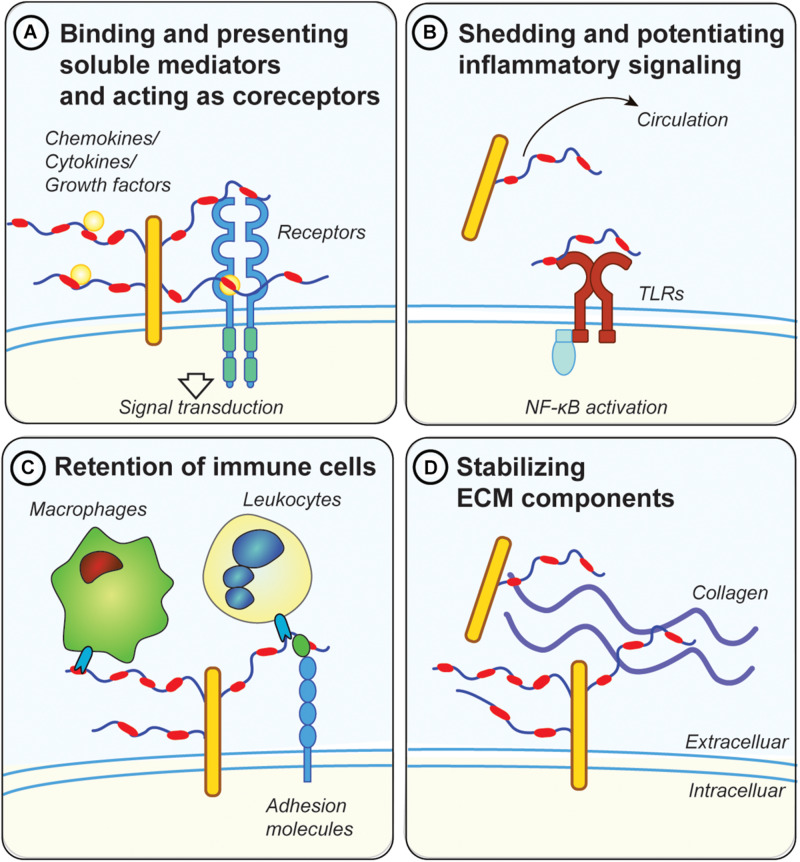
Possible mechanisms for proteoglycans in metabolic inflammation. **(A)** GAG chains, in particular HS, bind and present soluble inflammatory mediators, such as cytokines and chemokines at the cell surface. They also protect those factors from proteolytic degradation. Moreover, they act as co-receptors for ligand/receptor complexes, such as fibroblast growth factor 1 (FGF1) with FGF receptors. **(B)** Diabetes and metabolic inflammation lead to increased shedding of proteoglycans from the ECM, either by cleaving the protein core or the attached GAG chains. Shed proteoglycans and GAG chains have been shown to engage with toll-like receptors (TLRs), thereby potentiating the inflammatory response via NF-κB downstream signaling. Proteoglycans and GAGs released in the circulation can therefore have systemic effects and could be used as biomarkers for metabolic disease (e.g., GPC4). **(C)** Membrane bound proteoglycans (e.g., syndecans or glypicans) are in involved in retention of immune cells by directly engaging with lectins on the surface of immune cells. Proteoglycans also regulate the accessibility of adhesion molecules such as ICAM-1 on the cell surface which are important for the attachment of leucocytes. **(D)** Proteoglycans mediate the interaction between other ECM components such as collagens and fibrinogen. Dysregulations in ECM deposition lead to the development of fibrosis, a common pathology associated with metabolic disease.

HSPGs are a group of 17 family members of proteoglycans found in the basement membrane of cells. Despite their relatively small number of core proteins, they are structurally very diverse. Their primary GAG are HS chains reaching up to 40–300 sugar residues in length which can be modified by sulfotransferases on three different carbon positions (C2 on IdoA or C3 and C6 on GlcNAc) or an amine group (GlcNS) (Figure 1). Sulfation occurs in clusters of variable length generating heavily sulfated domains interspersed by unsulfated domains. A functional consequence of this molecular sulfation diversity is the formation of defined structural motifs which allow HS to bind and modulate the action of numerous specific extracellular ligands, such as cytokines and growth factors (39). HSPGs have many functions in inflammation, including: (i) building morphogen, growth factor, chemokine and cytokine gradients (Figure 2A); (ii) protecting chemo- and cytokines from proteolysis (Figure 2A); (iii) acting as co-receptors, for example with FGF receptors (FGFRs), to stabilize receptor/ligand complexes (Figure 2A); (iv) mediating signal transduction independently or by engaging inflammatory receptors such as toll-like receptors (Figure 2B); (v) regulating immune cell adhesion, migration, and activation (Figure 2C); and (vi) binding and regulating ECM components (Figure 2D) (40, 41). In addition, HSPGs play a central role in liver lipid homeostasis and thus influence hyperlipidemia and atherosclerosis development (42). Furthermore, HSPGs, such as perlecan and agrin play an essential role in the charge-mediated barrier of the glomerulus, important for the proper filtration of the kidney, and are associated with inflammation during kidney disease (43).

### Proteoglycan Biosynthetic and Modifying Enzymes in Meta-Inflammation

Due to the functional importance of GAG chains, enzymes that are involved in the assembly and functionalization of HS are consequential for HSPG-related processes making them important to consider in the context of obesity and meta-inflammation. The biosynthesis and modification of GAGs require several glycosyltransferases, sulfotransferases, and epimerases, all of which are well investigated. However, the regulatory circuits that impact their expression and activity in a spatial and temporal manner remain less clear.

Xylosyltransferases (XYLT) 1 and 2 initiate HS, CS and DS biosynthesis via catalyzing a covalent linkage of xylose to serine residues at the GAG-attachment site of the core protein (Figure 1). Given their critical role for GAG biosynthesis it is not surprising that their genetic inactivation results in gross phenotypic changes. Indeed, loss of XYLT1 in humans (and mice) is associated with short-stature due to bone development issues making it difficult to assess the impact in the context of meta-inflammation (44). Loss of XYLT2 in mice leads to decreased AT mass and local inflammation, concomitant with decreased glucose tolerance similar to a lipodystrophy phenotype (45). Also, *Xylt2*-deficient mice are afflicted by multi-organ dysfunction, making it difficult to assess to what extent the observed AT dysfunction plays an exclusive role in the observed metabolic effects (45). Nevertheless, it is important to consider the impact of *Xylt2* inactivation in diet-induced obesity.

HS undergoes extensive modifications beginning with *N*-deacetylation followed immediately by *N*-sulfation, a reaction catalyzed by *N*-deacetylase/*N*-sulfotransferases (NDST1-4). The structural complexity is further enhanced by epimerization and the sulfation of several positions by 2-*O*, 3-*O*, and 6-*O*-sulfotransferases. The sulfation reactions are non-template driven and require initial *N*-sulfation. Altered sulfation patterns influence the binding and signaling capacity of HS/ligand interactions (39) and compositional variation in sulfation within the population might influence their susceptibility for obesity and cardiovascular disease. An example of intrinsic variation of HS-sulfation are venous and aortic endothelial cells which differ in their degree of sulfation (46). This discrepancy might be the reason why there is increased leukocyte recruitment into small veins compared to capillaries and arteries (46). Global knock-out of *Ndst1* is perinatal lethal, but conditional knock-out mice allow investigating the impact of altered HS sulfation under various obesity- and inflammation-related conditions. In endothelial cells, inactivation of *Ndst1* inhibits granulocyte adhesion and diminishes binding of L-selectin *in vitro* (47) and results in reduced leukocyte recruitment in DKD *in vivo* (48). Embryonic stem cells from *Ndst1/2* double knock-out mice fail to differentiate into adipocytes *in vitro* (49) and decreased sulfation of macrophage HS through targeted deletion of *Ndst1* leads to increased atherosclerosis and obesity development driven by increased AT inflammation via type I interferon signaling (50, 51). However, to date no studies investigating the role of adipose NDSTs have been reported.

WNT and FGF binding to HS and hence their respective signaling modalities are regulated by HS, in particular via 6-*O*-sulfation (52, 53). Three different HS 6-*O*-sulfotransferases (HS6STs) having slightly different substrate specificities have been identified (54) and expression of *Hs6st1* is increased in macrophages from mice suffering from CVD and obesity (55–57). The importance of 6-*O*-sulfation in maintaining energy homeostasis has been evaluated in male *Hs6st2* knock-out mice. The systemic null mice present with increased weight gain and impaired glucose metabolism, even on a low-fat diet. Mechanistically, this was explained by reduced brown adipose tissue (BAT) mediated non-shivering thermogenesis as a result of reduced circulating thyroid hormone thyroxine (T4) levels that activate BAT (58). It is still unclear if this alteration in T4 levels is due to impaired uptake of the HS-binding thyroid hormone precursor thyroglobulin or due to the impact of HS on thyroid functionality to produce and secrete T4.

Heparanase (HPSE) is an extracellular HS degrading endo-β-D glucuronidase that is expressed in a variety of tissues. HPSE is involved in shedding of HSPGs from the ECM, which generates HS fragments ranging between 10 to 20 disaccharide units that remain biologically active. This leads to a re-organization of the ECM and therefore impacts cell motility and invasion (59). In an inflammatory context, this facilitates the recruitment of immune cells (60). HPSE activity also leads to upregulation of cytokine expression in macrophages (61, 62) and its expression in turn is induced by a variety of inflammatory cytokines, fatty acids (63) and high glucose (64). Diabetic patients often present with elevated HPSE in their circulation and urine (65) and *HPSE* upregulation is associated with DKD (66), as well as diabetes-associated cardiovascular diseases (67). Soluble HS fragments generated by HPSE have been shown to promote toll-like receptor (TLR) 4 signaling in dendritic cells (68) and human peripheral blood monocytes (69). Not surprisingly, infusion of mice with HS fragments resulted in marked pancreatic inflammation, while infusion in TLR4 knockout mice did not produce this inflammatory response (70). However, soluble HS fractions can also have protective functions and prevent bone marrow transplant rejection (71). It remains to be elucidated if HS fragments are released more prominently in metabolic dysfunctional patients and if their functions under obese conditions are beneficial or detrimental for infiltrating immune cells and the surrounding metabolic active cells.

Overall, investigation of HS-modifying enzymes comprises certain difficulties for the development of intervention strategies mostly due to their pleiotropic impact on all proteoglycans in every tissue, which makes it difficult to dissect and target the function of individual proteoglycans. Specific PGs have been implicated in metabolic homeostasis and inflammation and as such will be further discussed in the following sections.

### Syndecans – Major Hubs for Inflammation, Lipid Metabolism and Satiety Control

In mammalian cells, the Syndecan (SDC) family consist of four type I transmembrane HSPG (SDC1-4) (Table 1). They are expressed in a developmental and cell-type specific manner and involved in diverse biological processes ranging from morphogenesis to energy metabolism. The major functional groups of syndecans are the 1-3 attached HS chains on the N-terminus. They also carry 1-3 shorter CS chains closer to the plasma membrane. The number of attached GAG chains, their size, composition and sulfation pattern largely influence SDC’s binding capacity of its natural ligands. In this fashion syndecans bind and retain multiple heparan sulfate binding proteins (HSBPs). This will either attenuate or propagate HSBP functions, including properties of chemokines/cytokines and their interactions with leukocytes and endothelial cells (Figure 2) (31, 34). Several *in vitro* and *in vivo* studies have highlighted the diverse roles of SDCs in inflammation (31, 34, 72), but reports investigating the impact of SDCs on obesity-related metabolic inflammation are sparse (73–77). Using whole-genome linkage studies a SNP in chromosomal region 20q12-13, which contains the *SDC4* gene, has been linked with increased predisposition for T2D and obesity (78, 79). De Luca and coworkers showed in a small cohort that children homozygous for the minor *SDC4* SNP (rs1981429) allele presented with decreased lean mass and increased intra-abdominal fat mass (80). Fruit flies express one functionally conserved SDC isoform and its loss in female Drosophila reduced their body weight and body lipid content, while males did not show body weight changes. This was associated with reduced metabolic activity, measured by O_2_ and CO_2_ production, in mutant flies suggesting that SDCs play a key role in the regulation of body metabolism (80).

**TABLE 1:** Overview of proteoglycans associated with phenotypes in the context of metabolic dysregulation and meta-inflammation.

**Proteoglycan**	**Core mass (kDa)^a^**	**Chain type (number)^b^**	**Subcellular localization**	**Phenotypic observations**	
				
				**Humans**	**Relevant preclinical models**
**Heparan sulfate proteoglycans**					
Syndecan 1–4 (SDC1–4)	31–45	HS (2–3) in SDC2 and 4; HS/CS (3–4 HS/1-2 CS) in SDC1 and 3	Membrane-bound	Plasma SDC1 correlates with T1D and DKD (77) and hypertriglyceridemia in T2D patients (84); SNP in SDC4 linked with predisposition to T2D and obesity (78, 79, 80)	Increased atherosclerosis in *Apoe*^–^*^/^*^–^*Sdc1*^–^*^/^*^–^ mice (73); All SDCs: regulation of feeding behavior (89-92)
Glypican 1–6 (GPC 1–6)	57–69	HS (1–3)	Membrane-bound	Simpson-Golabi-Behmel syndrome (overgrowth) (GPC3-4) (98); GPC4 (serum, AT) increases with BMI, insulin resistance, NAFLD (100–106); GPC5 risk allele in DKD (107)	GPC5 correlates with DKD (108);
Perlecan (HSPG2)	∼470	HS (1–3)	Secreted/ECM	No data reported.	Obesity resistance in cartilage-rescued *Hspg2*^–^*^/^*^–^ mice (109); Role in lipoprotein retention in atherosclerosis (113, 117, 118)
**Chondroitin/Dermatan sulfate proteoglycans**					
Endocan (ESM1)	20	DS (1)	Secreted	Serum and AT levels increase in obesity (126, 134, 135); T2D (128, 129); atherosclerosis (130); DKD (131, 135); NAFLD (132); and psoriasis (133)	Correlation with DKD (136)
Decorin (DCN)	38–42	DS/CS (1)	Secreted/ECM	Increased expression in AT in obesity and T2D (143, 149, 151); Upregulated in DKD (156)	*Dcn*^–^*^/^*^–^ mice: increased obesity, AT inflammation, and glucose intolerance (151, 153); aggravated DKD (157)
Biglycan (BGN)	38–42	DS/CS (2)	Secreted/ECM	Upregulation in atherosclerotic plaques (166, 167); Increased in kidney injuries met with elevated inflammation, including DKD (171)	*Bgn*^–^*^/^*^–^ mice*:* reduced AT inflammation upon obesity (160); overexpression in mice promotes atherosclerosis (166, 167); *Bgn* accumulates in glomeruli of DKD mice (170)
**Keratan sulfate proteoglycans**					
Lumican (LUM)	∼37	KS (1)	Secreted	Liver expression correlates with severity of NASH and NAFLD (174–176)	*Lum*^–^*^/^*^–^ mice (females): increased obesity (177)
Osteoglycin (OGN)	25–72	KS and *O*-linked glycans	Secreted	OGN serum levels increase in response to weight loss in severely obese patients (184); Associated with atherosclerotic plaques (183)	Increased in atherosclerotic plaques in rabbits (182); Reduced levels of *Ogn* in obesity (185); *Ogn*^–^*^/^*^–^ mice: glucose intolerance and insulin resistance in diet-induced obesity (185)

*AT, adipose tissue; HS, heparan sulfate; DS, dermatan sulfate; CS, chondroitin sulfate; ECM, extracellular matrix; T2D, type 2 diabetes; NASH, non-alcoholic steatohepatitis; NAFLD, non-alcoholic fatty liver disease; DKD, diabetic kidney disease. ^*a*^Variation in core mass is due to species differences. ^*b*^Number of chains is based on the number of putative attachment sites for chain initiation as well as data from the literature; the actual number of chains varies by method, tissue, and species.*

Insulin promotes shedding of SDC1 ectodomains (81, 82) and increased inflammatory mediators and proinflammatory monocytes in patients with type-1 diabetes and nephropathy have been correlated with increased plasma SDC1 levels (77). Insulin also increases expression of SDC1 in a human hepatoma cell line but is downregulated by increasing fatty acids levels (83). In the liver, SDC1 is particularly important for TRL uptake from the circulation. Hence, reduced SDC1 expression or increased shedding contributes to the associated hypertriglyceridemia in T2D patients (84). In fact, T2D patients present with increased expression of an ECM enzyme, called sulfatase-2 (SULF2), that removes 6-*O* sulfate groups from HS chains on SDC1 and other HSPGs (85–87). Increased SULF2 expression was also observed in *db/db* mice, a diabetic and obese mouse model lacking leptin receptor expression. The increased liver SULF2 levels in *db/db* mice associated with reduced HS sulfation on hepatocytes accompanied by reduced SDC1-mediated TRL clearance. Moreover, therapeutic lowering of SULF2 using antisense oligonucleotides reduced hypertriglyceridemia in *db/db* mice (88) and introduced a new therapeutic window for T2D-associated hyperlipidemia.

SDC1 is also presented at the cell surface of macrophages where it influences migration and inflammatory resolution response (73). Expression of SDC1 differs between macrophage subclasses depending on their inflammatory profile. Resolving or M2 polarized macrophages express high levels of SDC1, in contrast pro-inflammatory M1-polarized macrophages completely lack SDC1 expression. In particular, macrophages derived from *Sdc1* knock-out mice have reduced motility of M2-resolving macrophages, which is associated with increased atherosclerosis development in *Apoe*^–/–^*Sdc1*^–/–^ mice fed a Western-type diet (73). This might be explained by SDC1 sequestering inflammatory and chemotactic mediators away from signaling receptors on macrophages to promote anti-inflammatory properties. Although these data have been observed in the context of atherosclerosis, it is likely that macrophage-expressed SDC1 impacts other metabolic complications such as obesity-induced diabetes.

All SDCs have been implicated in the regulation of feeding behavior by guiding neuronal development and plasticity (89–92). Energy intake is centrally regulated in the hypothalamus via orexigenic/pro-feeding (agouti-related protein, AgRP) and anorexigenic/anti-feeding, pro-opiomelanocortic neurons. Both types of neurons make contact with melanocortin-4 receptor (MC-4R) expressing neurons. Secretion of satiety hormones including α-melanocyte stimulating hormone (αMSH) and anti-satiety peptides such as AgRP and melanin concentrating hormone (MCH) lead to inhibition or stimulation of food intake, respectively (93). SDC3 is expressed in the hypothalamus and its cell surface levels are regulated by nutrient conditions. Fasting induces hypothalamic expression of SDC3 and its ectodomain is shed in response to feeding. *Sdc3*^–/–^ mice are resistant to diet-induced obesity (94) due to reduced food intake. Lack of SDC3 at the cell surface increases orexigenic signaling via AgRP by preventing engagement of the anti-satiety hormone MCH with MC-4R and potentiation of αMSH (90). During feeding, SDC3 is cleaved by metalloproteases inducing satiety via MCH/MC-4R signaling. Furthermore, the shedding process appears to be regulated under fasting conditions as well since a putative inhibitor of the shedding process, tissue inhibitor of metalloprotease−3, is increased by food deprivation. Transgenic overexpression of SDC1 in the hypothalamus promotes obesity due to increased food intake as overabundant SDC1 at the cell membrane interacts with AgRP to potentiate its orexigenic activity (91). Loss of SDC1 also induces hyperphagia, especially after fasting periods. However, this is a result of reduced intradermal adipogenic differentiation. Lack of insulating intradermal fat promotes cold-stress resulting in hyperphagia. However, this increased food intake does not promote weight gain since it meets the increased energy demand of BAT to maintain body temperature via enhanced non-shivering thermogenesis (95). Food intake was also decreased in *Sdc4*^–/–^ mice independent of the diet. Although unclear at this point which role SDC4 plays in feeding behavior, it further emphasizes the overall relevance of Syndecans in satiety control (89).

### Glypicans – Biomarkers for Metabolic Syndrome and Insulin Resistance

Glypicans (GPC) are a six-member family of cell surface glycosylphosphatidylinositol (GPI)-anchored HSPGs (96) (Table 1). GPC can be shed from the cell surface by phospholipase-mediated cleavage of the GPI-anchor (97) which gives them the potential not only to influence cell surface processes, but also processes in the extracellular environment and to act systemically (Figure 2). In humans, mutations in GPC3 and GPC4 are associated with the development of the Simpson-Golabi-Behmel syndrome (SGBS), an X-linked inherited overgrowth syndrome characterized by a broad spectrum of clinical manifestations, such as congenital, facial and cardiac abnormalities, organomegaly, and reduced viability primarily in male patients (98). Interestingly, pre-adipocytes from a male SGBS patient have been used as a model system for human adipogenic differentiation ever since their isolation in 2001 (99).

In an unbiased screen GPC4 was identified as an adipokine in a set of developmentally regulated genes that are differentially expressed in subcutaneous and visceral AT of mice and men (100). In healthy humans, subcutaneous AT has the highest GPC4 expression while in obese patients GPC4 expression decreases in subcutaneous AT and increased in visceral AT (100, 101). The most clinically relevant observation is a positive association between GPC4 expression in human white AT and both body-mass index (BMI) and central AT distribution. Since its discovery as a novel adipokine, several studies further confirmed this strong correlation using serum GPC4 levels and also identified that increased GPC4 serum levels positively associated with the prevalence of NAFLD and insulin resistance in at risk patients (101–106). When considering GPC4 as a biomarker for insulin resistance and NAFLD it is important to consider sex-specific differences as healthy men present with higher plasma GPC4 levels compared to women. However, circulating GPC4 levels in obese and insulin resistant female patients are dramatically elevated, reaching comparable levels as their male counterparts (101, 105). Collectively, GPC4 seems to be a potent biomarker for metabolic disease, however its exact functions in obesity development and metabolic inflammation remains to be fully established. Functionally, research supports the concept of GPC4 as an insulin sensitizer as GPC4 directly binds the insulin receptor, an interaction that is disrupted by insulin. Hence, it is plausible that the insulin receptor interaction with GPC4 stabilizes insulin receptor to prolong its presentation and insulin reception. Recombinant GPC4 administration increases insulin signaling in cultured adipocytes independent of its GPI anchor, but the importance of the HS chains for the GPC4-mediated insulin sensitization is unclear and remains to be determined (101).

GPC5, another GPI-anchored HSPG, associated in a genome-wide association study (GWAS) with acquired DKD [odds ratio 1.45 (95% CI, 1.18–1.79)] (107). The risk allele in the GWAS correlated with elevated GPC5 expression in podocytes. Similarly, GPC5 expression gradually increased in glomerular mesangial cells in murine T2D models and associated with progression and severity of DKD (108). In contrast, podocyte specific GPC5 knockdown in a preclinical FGF2 induced DKD model conferred resistance to podocyte and glomerular injury. These studies validate the importance of GPC5 in progression of DKD. It remains unclear how GPC5 influences the pathology, but it might involve sequestration of FGF2 and local modulation of FGF receptor activity (107, 108). Therefore, future studies are warranted to elucidate the involvement of GPC5 in DKD pathophysiology.

### Perlecan – Driver of Atherogenesis and Adipocyte Hypertrophy

The largest secreted proteoglycan (∼500 kDa) is Perlecan (HSPG2) carrying up to three HS attachments. It is an integral component of the ECM where it interacts with the basement membrane (109–113) (Table 1). HSPG2 is implicated in various pathological processes, such as tumor development, osteoarthritis, muscle hypertrophy, atherosclerosis and diet-induced obesity (113). Its physiological importance and complex biology is illustrated by the fact that a null-allele of *Hspg2* in mice is embryonic lethal, but can be perinatally rescued via transgenic expression of *HSPG2* in cartilage (111). Regulation of perlecan expression is intricate and modulated by many external factors including growth factors, chemokines, cytokines and cellular danger signals. Its expression and secretion is suppressed by interferon γ but activated by nuclear factor kappa-light-chain-enhancer of activated B cells (NF-κB) signaling (114, 115), as well as in macrophages by HIF1α and HIF1β upon hypoxia, a pertinent condition in atherosclerotic lesions and hypertrophic AT (116). HSPG2 is abundant in the arterial wall, specifically the intima. In this sub-endothelial layer of the artery HSPG2 binds and traps LDL via interaction of LDL-associated apolipoprotein B (apoB) and E (apoE) in an HS chain dependent manner. Knock-in mice expressing perlecan lacking the HS binding sites present with reduced subendothelial lipoprotein retention and atherosclerosis development (113, 117, 118). Hence, hypoxia-induced perlecan expression in macrophages could accelerate lipoprotein trapping in the subendothelial layers of arteries as well as promote hypertrophy (over hyperplasia) in expanding AT. It is thus not surprising that perinatally rescued *Hspg2*-deficient (*Hspg2*^–^*^/^*^–^tg) mice are resistant to diet-induced obesity due to reduced AT hypertrophy (109). The additional improvements in hepatic steatosis and insulin sensitivity might also be attributed to elevated muscle energy metabolism. However, the impact of *Hspg2*-loss on metabolic/adipose inflammation and insights as to how perlecan can affect adipocyte hypertrophy remain to be determined.

## Chondroitin, Dermatan and Keratan Sulfate Proteoglycans Have Pleotropic Roles in Meta-Inflammation Initiation and Progression

Unlike HSPGs, CSPG and DSPGs are a group of mostly secreted proteins forming an integral part of the ECM (Figure 1). They are involved in many essential physiological functions such as morphogenesis, inflammation and neuronal plasticity and play central roles in pathological processes such as cancer, osteoarthritis and thrombosis making them potential therapeutic targets (119, 120). Several secreted matrix CS/DS and KS proteoglycans contain leucine-rich repeats (LRRs) in their protein core (around 42 kDa), categorizing them as small leucine-rich proteoglycans (SLRPs). Five classes based on their structural relationship and associated GAG chain are described: Class I consists of CS/DS containing biglycan (Bgn), decorin (Dcn) and asporin, class II is formed by KS-associated PGs such as fibromodulin and lumican (Lum), class III consists of osteoglycin and opticin and non-canonical classes IV and V lack GAG attachments (121). SLRPs have been implicated in maintaining matrix assembly, thereby fine-tune the tissue-specific micro-environment and are involved in multiple metabolic processes, including inflammation (27).

The first step in CS/DS biosynthesis is building the shared GAG tetrasaccharide linker on a serine residue of the core protein (Figure 1). Subsequent polymerization, sulfation, epimerization and degradation of the disaccharide subunits requires a distinct set of enzymes not shared with HSPGs (122). Little is known about how alterations in CS/DS composition affect metabolic dysfunctions and meta-inflammation except for a report on a spontaneous mutated mouse line, small with kinky tail. This mouse model lacks functional chondroitin sulfate synthase 1 and presents with increased age-related, low-grade inflammation (123). Overall, this lack of knowledge is due to the fact that the number, diversity and redundancy of CSPG-specific biosynthetic enzymes make it difficult to probe this issue. Historically this has shifted the focus to evaluating core proteoglycans in meta-inflammation, which we will discuss in the following sections.

### Endocan – Potential Biomarker for T2D Severity and Co-morbidities

Transcription of the endothelial cell-specific molecule 1 (*ESM1*) gene produces Endocan, a DSPG that is secreted in the ECM carrying a single DS chain covalently linked to serine 137 (Table 1) (124). Primarily expressed by lung and kidney endothelial cells, endocan is upregulated by pro-inflammatory cytokines such as TNF and interleukin 1-β (IL1-β) (124). Once in the circulation ESM1 can interfere with leucocyte extravasation by blocking the interaction of leukocyte function-associated antigen-1 and intercellular adhesion molecule (ICAM-1), suggesting that endocan is part of a negative feedback loop to attenuate the inflammatory recruitment response (125). ESM1 is secreted by adipocytes and its expression progressively increases during adipogenesis. As expected AT *ESM1* expression and circulating ESM1 levels increased in obese patients (126). In contrast, insulin and cortisol administration inhibit ESM1 expression in adipocytes *in vitro* (126, 127). Because of its potential as a marker for endothelial dysfunction and its association with AT and obesity, endocan is evaluated as a potential biomarker for several obesity associated conditions. Several studies observed elevated plasma endocan levels in T2D patients (128, 129) as well as a correlation with the onset of T2D associated morbidities including atherosclerosis (130), nephropathy (131), and NAFLD (132). In psoriasis patients, a common chronic inflammatory skin disease, elevated endocan levels correlated with enhanced mean carotid artery intima-media thickness, BMI, and TNF levels (133). However, conflicting data in T2D patients indicated that using endocan as a prognostic biomarker is a much more sophisticated endeavor, as plasma endocan concentrations were significantly reduced and correlated inversely with waist circumference and CRP levels, a marker for systemic inflammation (126, 134, 135). In the same T2D patient population endocan serum levels associated with increased urinary albumin to creatinine ratios, suggesting a role for endocan in the progression of kidney injury in obesity-mediated diabetes (135). Evaluation of a DKD mouse model confirmed that ESM1 expression decreased in glomeruli with an ensuing reduction in endocan plasma levels and increased urinary concentration (136). Thus, evidence is mounting that endocan can be a useful biomarker for the severity of T2D and the onset of its co-morbidities. Collectively, functional studies of the role of endocan are needed to help to clarify the contribution of endocan to the development of T2D.

### Opposing Roles of Biglycan and Decorin in Obesity and Meta-Inflammation

Bgn and Dcn are ECM proteins with high expression in AT that share similar structural features (Table 1). Multiple roles have been attributed to Bgn and Dcn, including matrix remodeling via interaction with collagens (137), regulation of growth factor signaling such as transforming growth factor β (TGF-β) (138), and contribution to the proliferation of cells such as preadipocytes (139, 140). Both SLRPs regulate innate immune responses through direct interaction with TLR2 and TLR4 (Figure 2) (141, 142). However, despite their similarities they tend to have opposing roles in the context of diet-induced obesity (DIO) with Dcn having protective attributes and Bgn promoting meta-inflammation.

### Decorin – Friend or a Foe in Inflammatory Diseases?

One of the best studied SLRPs is DCN, an ECM CSPG that regulates both innate and adaptive immunity in opposing manner. DCN interacts with TLR2 and TLR4 on innate immune cells to promote expression of pro-inflammatory cytokines such as TNF and IL12p70. Support for its physiological importance as an enhancer of innate immunity was provided by the observation that LPS-induced sepsis is mitigated in *Dcn*^–^*^/^*^–^ mice (142). In contrast, DCN, as well as BGN, are inhibitors of adaptive immunity and specifically of classical complement activation. Both PGs bind and sequester complement component 1q thereby preventing its recruitment to antigen-antibody complexes. This event prevents proper C1 complex activation and suppresses the adaptive inflammatory response and cytokine production. This, in the context of metabolic inflammation, can attenuate complement overactivation at the onset of its manifestation (143, 144). DCN also modulates engagement of cytokines with their receptors. For example, DCN interacts with TGF-β, a major activator of fibrinogenesis, to suppress its response. The reduced TGF-β reception can further mitigate inflammation, fibrosis and tissue hypoxia in the context of metabolic disorders such as obesity and non-alcoholic steatohepatitis (NASH) (145).

Many factors, including cytokines, modulate DCN expression that result in upregulation, such as TGF-β and TNF (146), as well as down-regulation by IL-1, IL-6, and IL-10 (30, 147, 148). Its expression also exhibits regional variation, particularly between subcutaneous and visceral AT depots, with higher DCN expression in the latter (149, 150). DCN is predominantly expressed by the stromal vascular pre-adipocyte fraction and to a lesser extent by mature adipocytes (151). The complex transcriptional regulation also translates in a multifaceted impact of decorin expression on adipogenesis. Silencing of *Dcn in vitro* increased the differentiation potential of visceral preadipocytes without affecting subcutaneous preadipocyte differentiation. High levels of recombinant DCN protein was able to overcome this divergences as it inhibited adipogenic differentiation in both depots (150). It is generally well-accepted that subcutaneous and visceral ATs display distinct features such as different gene expression, higher lipolytic rate and decreased insulin sensitivity in visceral AT (152). Future research needs to elucidate if DCN is a key determinant of some of these depot-specific differences.

Obese and T2D patients present with increased *DCN* expression in AT. This phenomenon is attenuated after administration of thiazolidinediones, a potent class of insulin sensitizing drugs. This suggests that insulin resistance is partially responsible for the increase in AT *DCN* expression in T2D (143, 149). However, after bariatric surgery, which induces substantial weight loss and improved glucose tolerance, *DCN* expression is further upregulated in subcutaneous AT (151). Systemic *Dcn* knock-out mice have increased HFD-induced obesity, aggravated glucose intolerance and a higher risk of developing spontaneous intestinal tumors (151, 153). The study provided indirect evidence suggesting that altered DCN-associated ECM remodeling mediated some of these effects. In addition the lack of DCN production in white AT from HFD-fed *Dcn*^–^*^/^*^–^ mice associated with augmented AT inflammation measured by increased expression of complement and coagulation related genes (151). Other reports described DCN as a resistin receptor on adipose progenitor cells (140). Resistin is an adipokine promoting inflammation and insulin resistance in rodents (154) and in humans it is positively associated with AT macrophage content and increased during bouts of systemic inflammation (154). Single nucleotide polymorphisms (SNPs) in the human DCN gene locus correlate with elevated plasma resistin levels, while SNPs in the resistin gene correlate with higher susceptibility for T2D (155). It remains to be determined how and to what extend DCN impacts metabolic consequence associated with elevated resistin expression. One possibility is that DCN serves as a decoy receptor or scavenging agent which buffers the increased resistin secretion associated with excessive AT expansion.

In a patient study of DKD, both DCN and BGN were identified to be upregulated in kidney cortex and glomerular biopsies from patients with DKD. Only DCN was increased in the plasma of these DKD patients and correlated with a significant reduction in glomerular filtration rate (a clinical characteristic of DKD progression) (156). *Dcn* deficiency in a streptozotocin-induced type 1 diabetes mouse model promoted DKD, resulting in elevated albumin to creatinine ratios and increased fibrosis (157). Moreover, in a follow-up study, diabetic *Dcn*^–^*^/^*^–^ mice showed aggravated kidney injury met with an accumulation in renal BGN content, accentuating potential opposing roles of DCN and BGN in kidney injury (158). The protective effect of decorin in DKD is a multifactorial process and evidence supports that the impact is mediated via decorin binding to TGF and the insulin-like growth factor-I receptor. Binding to the former attenuates inflammation, while binding to the latter will promote anti-apoptotic effects in tubular epithelial cells, synthesis of fibrillin-1 in renal fibroblasts and inhibition of proliferation and migration. Decorin seems to have the potential to attenuate metabolic inflammation. Hence, factors that modulate its expression as well as decorin itself have great potential as future therapeutic targets for inflammation-associated morbidities in metabolic disease.

### Biglycan – Danger Signal in Metabolic Disease

Both biglycan and decorin are signaling molecules and established ECM-derived danger-associated molecular patterns (DAMPs). Under normal conditions BGN is sequestered in the ECM but gets released during cellular stress and inflammation, as for instance during obesity. Once in its soluble form BGN is a ligand for TLR2 and TLR4 present on innate immune cells. The complex between BGN, TLRs and their co-receptors cluster of differentiation (CD) 14 and lymphocyte antigen 96 promotes activation of an inflammatory cascade engaging the NLR family pyrin domain containing 3 (NLRP3) inflammasome leading to IL-1β and IL-6 secretion (141, 159). Because of its role as a DAMP, *Bgn*-deficient mice were studied to evaluate the impact of BGN on meta-inflammation in the context of diet-induced obesity. The lack of BGN expression in this HFD-fed model was associated with a reduction in obesity-driven inflammation in visceral AT independent of changes in adiposity (160). *Bgn* expression in AT reportedly increases during obesity which correlates positively with the expression of inflammatory genes and inversely with adiponectin (139, 160–163). The exact mechanism regulating *Bgn* expression during obesogenic conditions remains unknown, but involves factors such as pro-inflammatory cytokines (IL-6 and IL-1β) as well as adipokines such as adiponectin (141). Adiponectin prevents hyperglycemia and promotes fatty acid oxidation. Unlike most adipokines, plasma and AT adiponectin levels are reduced in obese patients and mice (164, 165). A correlation between biglycan and adiponectin expression is evident in *Bgn*-null mice as they have elevated adiponectin levels, independent of their diet. However, *Bgn* knock-down in adipocytes *in vitro* had the opposite effect on adiponectin expression (162). Thus, the mechanisms leading to the increased adiponectin expression *in vivo* might be a metabolic adaption and needs to be further studied.

In addition to its detrimental roles in obesity, BGN also influences atherosclerosis development. BGN is the proteoglycan that is most co-localized with apoB in murine and human atherosclerotic plaques (166, 167). When overexpressed in smooth muscle cells of mice lacking low-density lipoprotein receptor (LDLR), it promotes atherosclerosis likely because it enhances the retention of apoCIII-enriched LDL and TRLs in the subendothelial matrix (168, 169).

BGN has also been demonstrated to play a role in the development and progression of kidney disease in obese and diabetic experimental models. Thompson et al. demonstrated that *Ldlr* knockout mice which were induced to be diabetic through streptozotocin injections, experienced glomerular BGN accumulation. Interestingly, this was met with elevated renal lipid accumulation and an increase in TGF-β, suggesting the involvement of BGN in lipotoxicity-mediated DKD (170). Soluble BGN is being considered a biomarker for kidney injuries, which are met with elevated inflammation, including obesity-mediated DKD (171). Collectively, studies show that elevated BGN expression and shedding was associated with obesity associated co-morbidities, rendering BGN as a potential diagnostic marker and therapeutic target.

### Lumican – Pro-inflammatory Diagnostic Marker for NAFLD Progression?

Lumican (Lum) belongs to class II SLRPs that carry KS-chains (121). In contrast to HS, CS and DS which attach to the core protein at a serine residue, keratan sulfate is attached via *O*- or *N*-glyosidic bindings at asparagine and serine/threonine residues (Table 1 and Figure 1). Similar to BGN and DCN, Lum is found in the ECM where it interacts with collagens and is associated with repair processes in collagen-rich connective tissues (137). In contrast to BGN and DCN, Lum does not directly interact with TLR2/4, but interferes with the adaptor protein CD14 which facilitates LPS presentation to CD14 (172). Moreover, TNF-stimulated fibroblasts produce Lum that in turn promotes fibrocyte differentiation (173). In fact, Lum expression in the liver is tightly correlated with the severity of NAFLD and NASH and is currently evaluated as a biomarker for progression of such liver complications (174–176).

A recent report described a role for Lum in obesity and inflammation (177). As for other SLRPs, visceral AT exhibits higher Lum expression compared to subcutaneous AT and increases during obesity progression. Interestingly, Wolff and coworkers observed sex-specific differences in weight gain and obesity-related inflammation in Lum full body knock-out mice during diet-induced obesity (177). Based on its positive correlation with inflammation, loss of Lum should be protective against obesity and meta-inflammation. However, female *Lum*^–^*^/^*^–^ mice showed increased fat accumulation and AT inflammation which was also associated with accelerated progression of insulin resistance, whereas male mice were devoid of detrimental phenotypes (177). This study also tested the therapeutic potential of Lum by Adeno-associated Virus-mediated overexpression of Lum in male mice which mildly improved insulin sensitivity. As with other SLRPs, Lum’s role in obesity-related morbidities is controversial and could be based on the disease-specific context. Mechanisms explaining sex- and tissue-depending impacts of Lum pose interesting aspects for future investigations.

### Osteoglycin – Coordinator of Bone Formation With Novel Roles in Controlling Energy Homeostasis

Osteoglycin (OGN), also known as mimecan, is a class III SLRP associated with KS GAG chains. In addition to GAG chains other *O*-linked glycosylation of OGN have been described, but the glycan-type remains unidentified (Table 1). OGN is expressed in several isoforms resulting from differential splicing, alternative polyadenylation, and posttranslational modifications such as glycosylation (178). In an inflammatory context, the largest 72 kDa glycosylated leukocyte-derived isoform has been described to enhance the activation of TLR4 during viral cardiac inflammation (179). *Ogn* expression is upregulated by INF-y and TNF in an NF-κB dependent manner (180, 181). These phenomena could be connected to increased OGN expression found in atherosclerotic plaques, where these pathways are activated (182, 183). OGN binds and increases collagen cross-linking and bone formation, respectively. OGN is highly expressed in osteoblasts, but also expressed, albeit to a lesser degree, in cardiomyocytes, vascular smooth muscle cells, fibroblasts and neurons (178). Recent data shows that OGN is expressed in AT and involved in regulation of glucose homeostasis (184). Lack of *Ogn* expression increases glucose intolerance and elevates insulin levels in HFD-fed *Ogn*^–^*^/^*^–^ mice and ectopic administration of OGN improves glucose tolerance (185). Lee et al., showed that *Ogn* expression levels negatively correlate with diet-induced obesity and blood glucose levels and that ectopic administered OGN increases insulin secretion and glucose intolerance in mice. In severely obese humans, OGN serum levels increased in response to weight loss (185). It is assumed that this weight loss could be mediated by OGN regulating food intake. A previous study reported that injection of recombinant OGN in db/db mice induced an anorexic effect (184). However, this could not be reproduced in the later study of Lee et al., who showed the opposite effect (185). Different mouse models and technical differences might explain the discrepancies, but future studies are needed to clarify the effect of OGN on food intake.

## Clinical Significance and Potential Therapeutic Application of Proteoglycans

**FIGURE 3:**
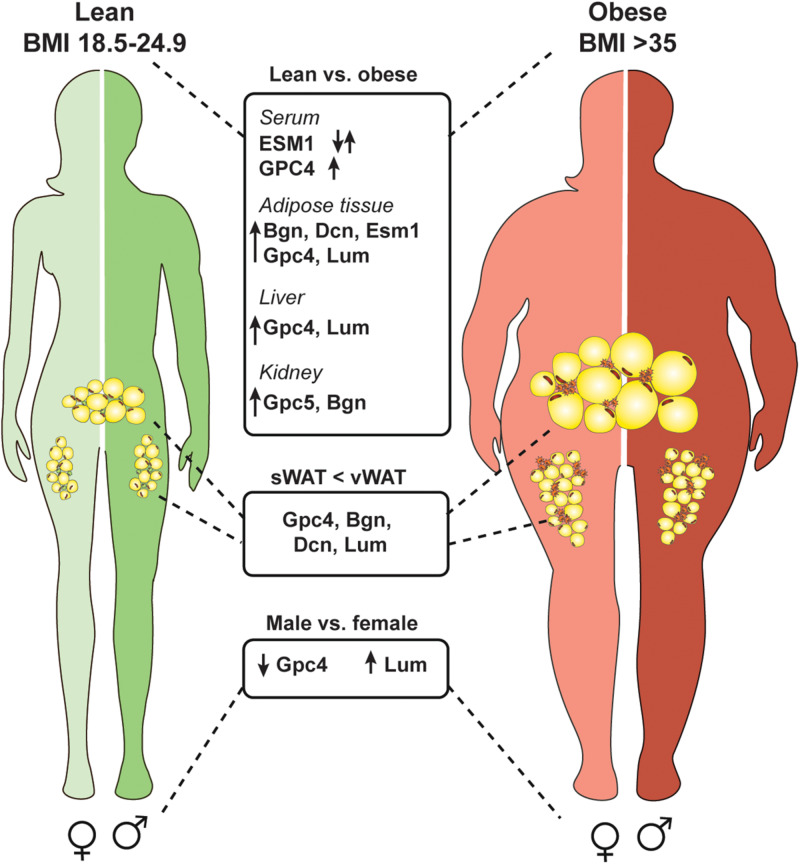
Several proteoglycans are deregulated in the obese state and differentially expressed in an adipose depot and sex specific manner. Obesity is defined by a body mass index of over 35 (weight in kg/height in m^2^). Multiple proteoglycans are upregulated in an obese state in adipose tissue, kidney, and liver. Also, serum levels of glypican-4 (GPC4) increase which could be used as a marker for insulin resistance. Serum endocan (ESM1) levels are deregulated in obese subjects and depending on the specific disease context can be up- or downregulated. Interestingly, some proteoglycans are higher expressed in visceral white adipose tissue (vWAT) compared to subcutaneous white adipose tissue (sWAT) which might account for depot specific differences (biglycan, Bgn; decorin, Dcn; lumican, Lum). Also, proteoglycans show differential expression in males and females independent of the diet.

Heparin, a naturally derived, heavily sulfated HS, is an essential and commonly used anti-coagulant in the clinic worldwide. Low molecular weight heparin analogs have anti-inflammatory properties without unwarranted anti-coagulant activity rendering these analogs as interesting candidates for clinical evaluation (186). Proteoglycans and associated GAG chains are utilized in the clinic for drug delivery methods and several experimental studies have evaluated the benefit of applying proteoglycans in the clinic as biomarkers and therapeutic targets for various diseases (187–189). This current review highlights several key clinical studies implicating proteoglycans as potential therapeutic targets or biomarkers for obesity-mediated inflammatory diseases. In summary, several proteoglycans are modulated in clinical and experimental models of obesity and its co-morbidities (summarized in Table 1 and Figure 3). Specifically, diabetic patients have been observed to have increased heparinase in the blood and urine, demonstrating that heparin and HS chain modifications could be important in diabetes and its co-morbidities (65). In separate studies authors demonstrated that soluble fragments generated from heparinase activity resulted in activation of TLR4 signaling, further implicating their involvement in meta-inflammation. Several studies mentioned above include strong association for many PGs and disease outcome in distinct patient cohorts of obesity and diabetes, such as GPC4 and ESM1. Interestingly, obese mice treated with oral administration of salmon cartilage proteoglycans experienced improvement in hyperglycemia and insulin sensitivity associated with a reduction in the expression of key inflammatory modulators such as TNF, IL-6 and C-X-C motif chemokine ligand 2 in AT (190). Studies like this one highlight the overall benefit of implementing the therapeutic potential of proteoglycans.

## Conclusion and Outlook

Undoubtful, proteoglycans play significant roles in mediating metabolic inflammation. Despite recent advances, our understanding of the specific roles of PGs during obesity progression and metabolic inflammation is still nascent. Many of the reports discussed in this review are primarily observational and lack mechanistic explanations. Model systems that allow studying proteoglycan interactions with inflammatory components have been generated in the past several years. However, few studies discriminate between the protein and GAG moiety of proteoglycans. This lack of properly addressing the importance of the glycoforms might explain discrepancies in study outcomes. Also, many of the proteoglycans’ functions might be triggered in a context- and tissue-dependent fashion. Thus, generation and investigation of conditional proteoglycan knock-out models is warranted to clarify the roles of proteoglycans upon diet-induced obesity and meta-inflammation. Overall it is evident that proteoglycans are interesting diagnostic or therapeutic targets, and specific roles in obesity-related inflammation and receptor interactions need to be fully identified and understood prior to consider them as such in the future.

## Author Contributions

PG and AP: conceptualization, writing – review and editing, and funding acquisition. AP, GD, and PG: writing – original draft. AP: Visualization; PG: supervision and project administration.

## Conflict of Interest

The authors declare that the research was conducted in the absence of any commercial or financial relationships that could be construed as a potential conflict of interest.

## Abbreviations

α MSH: A -melanocyte stimulating hormone
AgRP: agouti-related protein
ApoB: apolipoprotein B
ApoE: apolipoprotein E
AT: adipose tissue
BAT: brown adipose tissue
Bgn: biglycan
BMI: body-mass index
CD: cluster of differentiation
CRP: C-reactive protein
CSPG: chondroitin sulfate proteoglycans
DAMPs: danger-associated molecular patterns
DCN: decorin
DIO: diet induced obesity
DKD: diabetic kidney disease
DSPG: dermatan sulfate proteoglycans
ECM: extracellular matrix
ESM1: endothelial cell-specific molecule 1
FGFs: fibroblast growth factors
GAG: glycosaminoglycan
Gal: galactose
GlcA: glucoronic acid
GlcNAc: *N*-acetyl glucosamine
GlcNS: *N-*sulfated glucosamine
GPC: glypicans
GPI: glycosylphosphatidylinositol
GWAS: genome-wide association study
HFD: high fat diet
HIF1: hypoxia inducible factor 1
HPSE: heparanase
HS: heparan sulfate
HS6ST: HS 6-*O*-sulfotransferase
HSPG: heparan sulfate proteoglycans
HSPG2: perlecan
ICAM-1: intercellular adhesion molecule
IdoA: iduronic acid
IL-1 β: interleukin 1 beta
IL-6: interleukin 6
KS: keratan sulfate
KSPG: keratan sulfate proteoglycans
LDL: low-density lipoproteins
LDLR: low-density lipoprotein receptor
LPS: lipopolysaccharide
Lum: lumican
MC-4R: melanocortin-4-receptor
MCH: melanin concentrating hormone
NAFLD: non-alcoholic fatty liver disease
NASH: non-alcoholic steatohepatitis
NDST: N-deacetylase/N-sulfotransferase
NLRP3: NLR family pyrin domain containing 3
OGN: osteoglycin
PGs: proteoglycans
SDC: Syndecan
SGBS: Simpson-Golabi-Behmel syndrome
SLRP: small leucine-rich proteoglycans
SNPs: single nucleotide polymorphisms
SULF2: sulfatase 2
T1/2D: type-1/2 diabetes
T4: thyroxine
TGF- β: transforming growth factor beta
Th1: T-helper type 1
TLR: toll-like receptor
TNF: tumor necrosis factor
TRL: triglyceride-rich lipoproteins
VLDL: very low-density lipoproteins
XYLT: xylosyltransferases.
